# Impact of daily administration of blackberry extract on gerbil model of transient cerebral ischemia

**DOI:** 10.1590/acb397424

**Published:** 2024-09-09

**Authors:** Asahi Oda, Kazuhisa Sugai, Masahiko Fujisawa, Yoji Hakamata, Shou Kobayashi, Eiji Kobayashi

**Affiliations:** 1 Nippon Veterinary and Life Science University – School of Veterinary Nursing and Technology – Department of Basic Science – Tokyo – Japan.; 2 Nippon Veterinary and Life Science University – School of Veterinary Nursing and Technology – Research Center for Animal Life Science – Tokyo – Japan.; 3 Kobayashi Regenerative Research Institute, LLC – Administrative Department – Wakayama – Japan.

**Keywords:** Plant Extracts Blackberries, Ischemia, Brain, Neurons, Gerbillinae

## Abstract

**Purpose:**

Blackberries are rich in polyphenols and are a human health food continuously consumed to improve health and reduce diseases caused by aging. Herein, we evaluated the effects of daily blackberry administration before and after transient cerebral ischemia in gerbils.

**Methods:**

Blackberry extract (BBE) was orally administered twice a day for two weeks to protect against ischemic events during continuous administration. On the seventh day after administration, the bilateral common carotid arteries were transiently occluded for 5 min. To verify its therapeutic effect, BBE was administered after ischemia using a similar protocol without pre-administration. In both experiments, the number of viable neurons in the CA1 region of the hippocampus was assessed seven days after ischemic treatment.

**Results:**

The number of neurons in the group treated with BBE before ischemia was higher than that in the group treated with distilled water (*p* = 0.0601), and similar to that in the control group. In the BBE administration experiments after ischemia, the number of neurons was significantly reduced compared to that in the control group (p < 0.0001).

**Conclusions:**

Continuous BBE intake is expected to prevent or ameliorate ischemic events such as transient cerebral ischemia.

## Introduction

Berries have attracted attention as health food for humans because they contain many polyphenols, vitamins, minerals, fiber and health benefits^1^^,^ 2. Recently, clinical trials using raspberries have been conducted in the field of beauty, showing that facial skin color, elasticity, radiance, smoothness, scaliness, and wrinkles in 50-middle-aged women significantly improved^3^. Large-scale clinical trials are required to demonstrate the effects of health foods on humans and the preventive administration of beauty products on adverse events. However, in highly individualized human studies, it is costly and time-consuming to determine the range of efficacy^4^^-^ 6.

Numerous animal studies have demonstrated that polyphenols, which are abundant in blackberry (BB) fruit and seeds, have antioxidant^7^^,^ 8, antitumor^9^^,^ 10, anti-aging^3^, anti-inflammatory^11^^,^ 12, anti-lipid^13^, and anti-stress effect^s14^. In many of these reports, to evaluate the effects, experimental impairment models have been established, and administration has been initiated at the same time or after impairment^15^. However, it has recently been reported that pretreatment with BB leaf extract for two weeks improves hepatic ischemia-reperfusion injury in rats^16^. We previously reported that repeated consumption of BB improves hyperlipidemia and that ingestion before the onset of adverse events is beneficial^17^.

In this study, we aimed to evaluate the effects of daily intake of BB as a human health food in a gerbil model of transient cerebral ischemia that may occur incidentally in older people. Cerebral ischemia is an intractable disease that causes nerve cell death and motor and higher brain dysfunction. Although various animal models have been developed to elucidate the pathogenesis of cerebral ischemic injury, it can be induced in gerbils simply by occluding the common carotid artery, owing to the incomplete construction of the basilar artery, and the degree of injury can be selectively induced from neuronal cell death to cerebral infarction by the occlusion time of the vessels^18^. Considering that the composition of BB depending on the production area and picking season^19^, we used BB from the main production areas of Japan.

## Methods

### Animals

Mature male (n = 12) and female (n = 12) Mongolian gerbils (*Meriones unguiculatus*) (body weight, 70–130 g) bred in our laboratory were used in this study. All animals were kept in our laboratory and housed in an environment with 23 ± 2 °C, humidity of 55 ± 10%, and a 14-h/10-h light/dark cycle, and were provided solid mouse/rat feed (EF solid feed; Oriental Yeast Co., Ltd., Tokyo, Japan) and water *ad libitum*.

This study was approved by the Institute Animal Care and Use Committee (Nippon Veterinary and Life Science University, Tokyo, Japan; Permit No. 2023K-72) and followed the Nippon Veterinary and Life Science University guidelines for the care and use of laboratory animals. All sections of this study adhered to the ARRIVE guidelines for animal research^20^.

### Preparation of blackberry extract and measurement of polyphenol concentration

**Figure 1:**
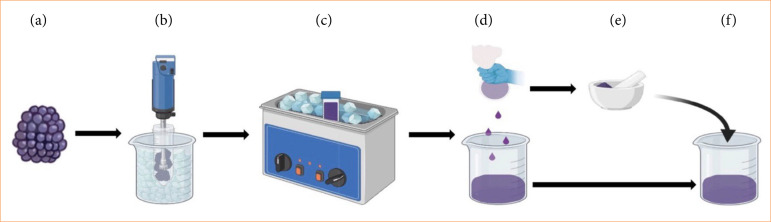
Extraction process of blackberry extract (BBE). **(a)** Blackberry. **(b)** Blackberries were placed in a centrifuge tube and homogenized on ice. **(c)** Blackberries were sonicated. **(d)** Sample was packed into gauze and squeezed. **(e)** Residues were dried and ground to powder. **(f)** Powder was mixed with squeezed liquid to create BBE.

Source: Elaborated by the authors.

BB used in this study were obtained from Amanogawa Co. (Aomori, Japan). Frozen BB were thawed in a water bath at 37 °C, homogenized using polytron-aggregate (PT1200E, Kinematica Co., Malters, Switzerland), and liquefied by sonication for 30 min using a US-Cleaner (US-2R, SND Co., Nagano, Tokyo). Liquified BB were pressed with gauze to separate the seeds, skin, and juice. The seeds and skin were dried in a dryer (NDS-520, Wakenyaku Co., Kyoto, Japan) and ground into powder in a mortar (Asvel Co., Nara, Japan). The seed and skin powders were placed in separated BB juice, dissolved in a water bath at 55 °C, and the resulting solution was regarded as BB extract (BBE) (Fig. 1). The polyphenol content of BBE was measured using the Folin–Ciocalteu method (polyphenol content: 280 mg/100 g BBE). Distilled water (DW) was used as a reference material for BBE.

### Blackberry extract administration schedule and cerebral ischemia procedure

The animals were randomly divided into three groups: control (n = 6), DW (n = 8), and BBE (n = 10).

#### Pre-ischemia blackberry extract administration group

**Figure 2:**
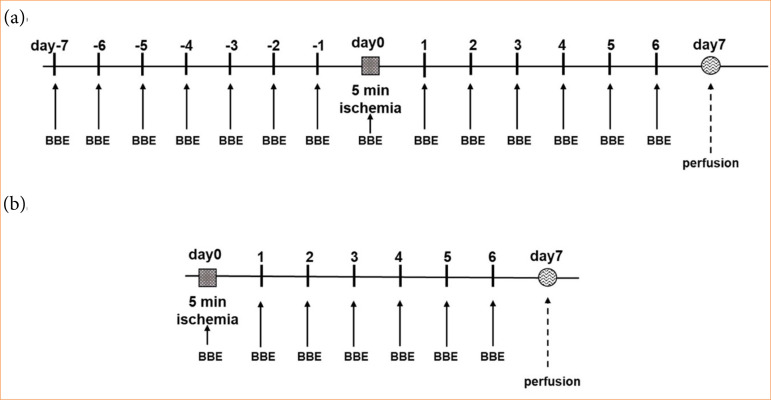
Blackberry extract (BBE) administration and brain ischemic operation protocol. **(a)** Pre-ischemia administration protocol. BBE was orally administered twice a day from seven days before ischemia. BBE administration was continued for seven days after ischemic operation. On the postoperative day 7, the brain was fixed via transcardial perfusion. **(b)** Post-ischemia administration protocol. BBE was orally administered twice a day for seven days after ischemic operation. On the postoperative day 7, the brain was fixed via transcardial perfusion.

Source: Elaborated by the authors.

Animals were divided into DW (n = 5) and BBE (n = 4) groups. Animals were orally administered DW or BBE with a probe at the dose of 10 mL/kg bodyweight twice a day (10 a.m./5 p.m.) under light isoflurane inhalation anesthesia seven days before cerebral ischemia (Fig. 2a).

#### Post-ischemia blackberry extract administration group

Animals were divided into DW (n = 3) and BB (n = 6) groups. After reperfusion following cerebral ischemia, the animals were orally administered DW or BBE with a probe at the same dosage as the pre-treatment groups twice a day for three (n = 3) or seven days (n = 3) (Fig. 2b).

Cerebral ischemia was induced under isoflurane inhalation anesthesia (induction at 5%, maintained at 2%). Animals were placed on a hot plate maintained at 37°C (KN-475-3-40, Natsume Seisakusho Co., Tokyo, Japan), and bilateral common carotid arteries were occluded as previously described21. Briefly, under anesthesia, a midline incision was made in the neck, and the bilateral common carotid arteries were exposed. Both common carotid arteries were clamped for 5 min using artery clips (AS1-40; Bear Medic Co., Ibaraki, Japan). After 5 min of occlusion, the clips were removed to resume blood flow, and the incised skin was closed using wound clips (Becton Dickinson Japan Co., Tokyo, Japan). The animals in the control group underwent the same procedure as the ischemic animals, except that the bilateral common carotid arteries were not occluded. No solution was administered to the control group.

### Assessment of cerebral neurons

To assess the effect of BBE on brain ischemic neuronal cell death, all animals used in this study were perfusion-fixed. Seven days after ischemia-reperfusion, the animals were deeply anesthetized with an intraperitoneal injection of pentobarbital sodium (65 mg/mL, 1 mL/kg bodyweight). The transcardial approach was used to perfuse the brains with 100 mL of heparinized saline followed by 100 mL of 10% phosphate-buffered formalin. The brains were fixed overnight in 10% phosphate-buffered formalin. The fixed brain was coronally sectioned at the pituitary level, embedded in paraffin and cut into 5-µm sections, and stained with hematoxylin and eosin (HE). Each brain section was scanned with a NanoZoomer-SQ 40× mode (0.23 µm/pixel) scanner (Hamamatsu Photonics K.K., Shizuoka, Japan), and images were analyzed by NDP.view 2 software (Hamamatsu Photonics K.K.) to measure the number of neurons in the hippocampal CA1 area. Neurons with distinct nuclear membranes and clear nucleoli were defined as viable neuronal cells and counted.

### Statistical analysis

All data are presented as mean ± standard deviation. All statistical analyses were conducted using GraphPad Prism version 10.1.1. Comparisons were performed using one-way analysis of variance and Tukey’s multiple comparison test for post-hoc analysis. Statistical significance was set at *p* < 0.05.

## Results

### Effect of pre-ischemic administration of blackberry extract

**Figure 3:**
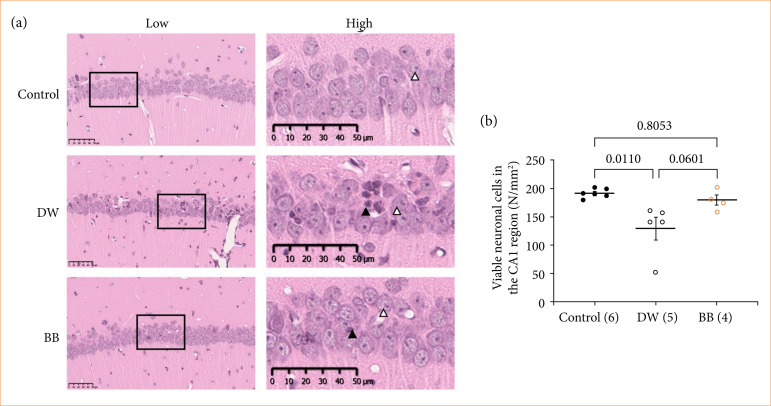
Effect of pre-ischemia administration of blackberry extract (BBE). **(a)** Images of ischemic CA1 region of hippocampus stained with hematoxylin and eosin. The image on the right is an enlarged view of the image on the left. **(b)** The number of viable neuronal cells in the CA1 region. The numeric values indicate p-values between each group.

The number of viable neuronal cells in the CA1 region of the hippocampus after transient cerebral ischemia in gerbils orally administered BBE for seven days is shown in Fig. 3. Remarkably, the number of viable neurons in both groups subjected to cerebral ischemia was abundant despite cerebral ischemia. The number of viable neurons in the DW group was significantly lower than that in the control group (*p* = 0.0110). The number of viable neuronal cells in the BBE group tended to be higher than that in the DW group, although there was no significant difference between the two groups (*p* = 0.0601), which was equivalent to that in the control group (*p* = 0.8053).

Δ: typical viable neuron; ▲: dead neuron. Source: Elaborated by the authors.

### Effect of post-ischemic administration of blackberry extract

**Figure 4:**
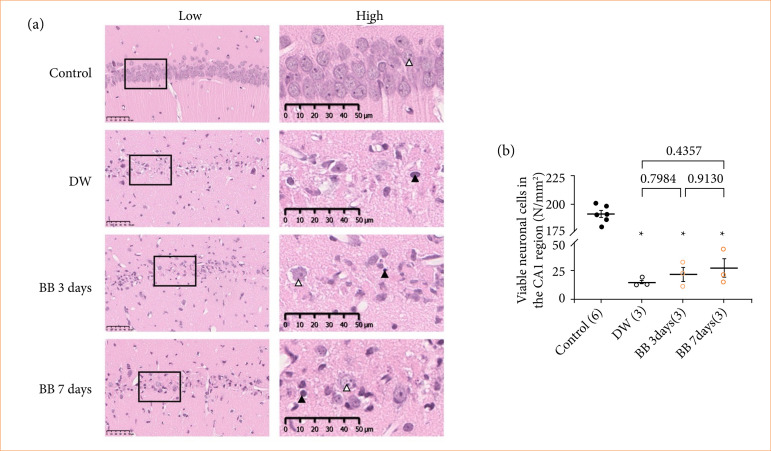
Effects of post-ischemia administration of blackberry extract (BBE). **(a)** Images of ischemic CA1 region of hippocampus stained with hematoxylin and eosin. The image on the right is an enlarged view of the image on the left. **(b)** The number of viable neuronal cells in the CA1 region. The numeric values indicate p-value between each group.

The number of viable neuronal cells in the CA1 region of the hippocampus of gerbils that repeatedly underwent BBE administration after cerebral ischemia is shown in Fig. 4. The number of viable neurons in both groups subjected to cerebral ischemia was significantly lower than that in the control group (*p* < 0.0001). Although the number of viable neuronal cells in the BB seven days group tended to be higher than that in the BB three days group. The number of cells in both groups did not significantly differ from that in the DW group.

Δ: typical viable neuron; ▲: dead neuron; *p < 0.0001 vs. control group. Source: Elaborated by the authors.

## Discussion

The BB examined in this study contain anthocyanins, other phenolic compounds, flavanols, and ellagitannins, which contribute to their high antioxidant potency and other biological activities, and are consumed worldwide as a human health food^3^. However, large-scale randomized controlled studies are required to verify these effects in humans. In this study, we evaluated the efficacy of repeated administration of BBE on cerebral ischemia in gerbils.

In the two-week pretreatment experiment, BBE consumption dramatically reduced neuronal cell death in the CA1 region of the hippocampus with transient cerebral ischemia. The number of neurons was equivalent to that of the control group, which was not subjected to cerebral ischemia. Repeated intake of polyphenols has been reported to reduce subsequent cerebral ischemic injury in other experimental systems^22^^,^ 23. This phenomenon is known as preconditioning and is anticipated to be a new therapeutic approach for stroke in humans through healthy food consumption^24^.

The prominent effect of the two-week BBE administration in this study may have induced not only an improvement in the lipid system before the event, but also this preconditioning^17^. In the pretreatment experiment, the number of viable neurons in the BBE group showed little variation and no decrease, whereas the DW group showed a large variation among individuals. Even in the DW group, half of the animals had abundant remnant nerve cells that appeared to be resistant to ischemia. For pretreatment with BBE and DW, oral administration was performed twice a day under isoflurane inhalation anesthesia.

This anesthetic treatment may temporarily paralyze nerves and induce a decrease in blood pressure and reflexes. In some animals in the DW group, this stress may have induced a preconditioning phenomenon that reduced the cerebral ischemia-reperfusion injury. Experimental evidence of such preconditioning phenomena has been reported in the brains^25^ and livers^26^ of rat models of ischemia. Mild stress stimulates the adrenal glands, and the glucocorticoids secreted by the adrenal glands are thought to act on the brain and induce anti-inflammatory effects^27^. However, although numerous studies have been conducted over a long time, the comprehensive mechanism of ischemic preconditioning is still controversial, and much remains unclear^28^^,^ 29.

In therapeutic administration, BBE treatment after cerebral ischemia did not suppress neuronal cell death. Although it has been reported that polyphenols contained in BB have antioxidant effects and rescue neuronal cell death^7^^,^ 8, it remains unclear whether the results of post-administration in this study were due to insufficient BBE components or whether the ischemic injury in this model was too high.

The BB used in this study were sourced from Aomori, which has the highest production rate in Japan. Although the freeze-drying^8^ and acetone methods^12^ are common methods for extracting BB, we focused on the inclusion of all components in consideration of their generality and simplicity as health food. Frozen BB were homogenized and sonicated, and the remaining seeds and peels were ground and dissolved. The concentration of polyphenols in the extract obtained by this method was comparable to that reported previously, suggesting no discernible differences due to the extraction methods^19^.

## Conclusion

This study demonstrates that continuous prophylactic intake of BBE stably suppresses neuronal cell death in the CA1 region of the hippocampus after transient cerebral ischemia. These results suggest that the continuous consumption of BB as a health food may be effective in developing tolerance to cerebral ischemic injury.

## Data Availability

Raw data were generated at Nippon Veterinary and Life Science University. Derived data supporting the findings of this study are available from the author Hakamata Y upon reasonable request.
